# Dynamic CRRT Prescription for Complicated Critically Ill Patient: A Case Report

**DOI:** 10.1155/2024/1837150

**Published:** 2024-11-20

**Authors:** Clemente Santorsola, Alberto Corona, Matteo Cecchi, Niccolò Castellani Nicolini, Elena Zendra, Alice Capone, Ivan Gatti, Matteo Brivio, Silvia Falsini, Gianluca Villa

**Affiliations:** ^1^Accident and Emergency Department and Anesthesia and Intensive Care Medicine Department, ASST Valcamonica, Esine Hospital, Via Alessandro Manzoni, 142, Esine 25040, Italy; ^2^Department of Health Sciences, Section of Anesthesiology, Intensive Care and Pain Medicine, University of Florence, Florence, Italy; ^3^Department of Anesthesia, Intensive Care and Transplant, Azienda Ospedaliero Universitaria Pisana, Pisa, Cisanello, Italy; ^4^Department of Anesthesia, Critical Care and Emergency, Spedali Civili University Hospital, Brescia, Italy; ^5^Anesthesia and Cardiac Intensive Care, Department of Critical Care and Emergency, Papa Giovanni XXIII Hospital, Bergamo, Italy; ^6^Department of Anesthesia and Intensive Care, Azienda Ospedaliero Universitaria Careggi, Florence, Italy

**Keywords:** acrylonitrile membrane, acute kidney injury, citrate, CRRT, CVVHD, CVVHDF, cytokines, high cut-off membranes, oXiris membrane

## Abstract

Continuous renal replacement therapies (CRRTs) and sequential extracorporeal blood purification (EBP) therapies can be used in patients with severe COVID-19 disease to support kidney failure and restore immune homeostasis. EBP prescription should be based on the patient's clinical needs and frequently re-evaluated during the intensive care unit (ICU) stay. Personalization of treatment at the bedside plays a fundamental role for patient recovery. This aim can be simplified by using both clinical and molecular data collected from a patient-individualized web registry. In this case report, we describe how we apply a sequential approach to EBP therapies following the rapid evolution of a critically ill COVID-19 patient with acute kidney injury. We show patient strategies and outcomes using bedside data from a registry-based method for the routine use of EBP. We explain the choice of specific hemofilter prescription, also focusing on dose and anticoagulation strategies. We describe the difficulties, uncertainties, and mistakes made during EBP prescription. Furthermore, we discuss the causes and workable solutions that can be adopted by the ICU physician for a better EBP prescription, considering the current lack of well-recognized indications.

**Trial Registration:** ClinicalTrials.gov identifier: NCT03807414

## 1. Introduction

COVID-19 is a complex disease caused by SARS-CoV-2 infection, frequently associated with the development of acute kidney injury (AKI) [1–3].

Continuous renal replacement therapies (CRRTs) and the broader concept of extracorporeal blood purification (EBP) can be prescribed in patients with COVID-19 and AKI to support kidney function and interact with the pathophysiology of the dysregulated host immune response caused by SARS-CoV-2 infection [3]. The probability of success of the available blood purification methods is increased by individualized therapy and the selection of treatment candidates based on clinical and molecular characteristics. The prescription should be made initially by considering the purpose of treatment and the clinical goal to be achieved. Thus, the evolution of the patient's clinical condition should dynamically guide the physician's strategy when dealing with complex clinical scenarios such as the one presented here. The ICU physician must choose the appropriate filter and anticoagulation strategy to provide an adequate dose of extracorporeal clearance. These characteristics must be carefully considered during the prescription phase to tailor treatment to the patient's needs. Several critically ill patients, including those with COVID-19, have unique clinical needs that make the prescription and administration of EBP a challenging phase, where knowledge of membrane characteristics can be of crucial importance [3]. ICU physicians are required to decide on timely and appropriate treatment strategies to fulfill the patient's needs at the bedside; nonetheless, practical guidelines or specific suggestions on the timing and prescription of EBP are lacking in literature. Prescriptions are often uncertain, and perceptions of patient outcomes are often misleading.

This case report describes a real-life approach to EBP therapy applied to a complex case of a critically ill COVID-19 patient with multiple organ dysfunction. We perform a self-critical and analytical assessment of our bedside practice, specifically the adoption of EBP, outlining the rationale and stated goals when prescribing sequential extracorporeal therapies, and our difficulties in monitoring and delivering the prescribed treatments. Overall, we highlight the lack of strategies available today to objectively monitor EBP at the bedside, to promote precision medicine in this field, and to encourage self-assessment of EBP and practice.

## 2. Case Presentation

We present the case from the ARRT registry (Ethics Committee of Brescia NP 4679, http://www.arrt.eu/) of a 60-year-old man admitted to the emergency department for COVID-19. He developed upper respiratory tract symptoms and fever 7 days before presentation. On admission, the patient had respiratory distress and a peripheral oxygen saturation (SatO_2_) of 88%–90% on room air, 94% with a nonrebreathing mask at 15 L/min oxygen. Chest CT scan shows an extensive bilateral peripheral ground glass infiltrate. Two days later, the patient suddenly worsens; he is intubated and admitted to the ICU for mechanical ventilation and pronation cycles. The patient received steroid therapy with methylprednisolone 120 mg/daily for 8 days on a staged basis; he was also treated with Pentaglobin 5 g/daily for 7 days. The patient also received immunosuppressive treatment with hydroxychloroquine and antiretroviral treatment with lopinavir/ritonavir for a total of 6 days in the initial phase of hospitalization. Parameters showing multiorgan function and related extraorgan support in the first 2 weeks after ICU admission are described in Table 1.

During the first few days in intensive care, his clinical conditions rapidly worsen by developing hemodynamic shock (norepinephrine infusion: 0.15 mcg/kg/min), liver failure (bilirubin 4.29 mg/dL), and kidney dysfunction. Serum creatinine is above 2.5 mg/dL (2x from baseline); oliguria occurs despite continuous furosemide infusion (dose: 2.5 mg/h). Stage 2 AKI was diagnosed according to the KDIGO criteria. Stress test with furosemide [4] is negative, we consequently expect fluid overload. This patient also becomes hypoxemic and hyper inflamed. On the fifth day of ICU admission, because of all these conditions, awaiting the positive blood culture, we started EBP with a high cutoff polyarylethersulfone membrane with a surface area of 1.1 m^2^ (SepteX, Baxter, Deerfield, IL, USA) under continuous venovenous hemodialysis (CVVHD) with the aim of ultrafiltration and removal of proinflammatory cytokines. The high treatment efficiency guaranteed by increased membrane porosity allows this strategy to support kidney function and simultaneously remove bilirubin and large molecules (e.g., cytokines), even in CVVHD modality. The prescription settings are presented in Table 2.

Notably, this specific membrane cannot be used with isotonic citrate solutions for regional citrate anticoagulation (RCA) for safety reasons [1]. Systemic infusion of heparin as an anticoagulation strategy is therefore opted for. Unfortunately, treatment was discontinued after 18 due to clotting. The inadequacy of this anticoagulation strategy in a hyperinflamed patient has been indicated as the main trigger for clotting activation. This is in line with the results of a previous study which shows that higher hematocrit values before EBP and the absence of systemic anticoagulation strategies are independent predictors of circuit clotting. Indeed, patients' hematocrit values before EBP were 35.9%, higher than the average hematocrit (35%) in the study's clotting group. The treatment strategy is therefore modified and EBP is performed with a highly biocompatible high-flux membrane with advanced nonselective adsorption properties (oXiris membrane, Baxter, Deerfield, IL, USA) in continuous venovenous hemodiafiltration (CVVHDF). The oXiris membrane is an acrylonitrile-polyethyleneimine–based high-flux hemodiafilter with a heparin-coated surface (membrane surface area: 1.5 m^2^). The rationale behind this strategy assumes that the heparin coating is locally bioactive and that its effect is clinically relevant in reducing premature clotting.

Furthermore, as serum lactate and bilirubin levels are stable, an RCA with low citrate load is used despite acute liver failure. This approach is performed with strict monitoring of the liver function and lactate trend [5]. Treatment lasts 72 h without clotting events and with stable patient serum lactate levels; transmembrane pressure (TMP) and venous pressure are normal. The patient's clinical condition improves during treatment, especially regarding systemic inflammation and hemodynamic stability. After 72 h, renal support is still necessary due to persistent oliguria and resistance to furosemide infusion. Therefore, an acrylonitrile sodium methyl sulfonate copolymer high-flow hemodiafilter (AN69ST150, Baxter, Deerfield, IL, USA; membrane surface area: 1.5 m^2^) is prescribed in CVVHDF and RCA. Prescription settings are presented in Table 2. Currently, immunomodulation is excluded from the treatment goals, a standard high-flux membrane is thus adopted to exclusively support renal function. Unfortunately, membrane clotting occurs after 40 h. Previous experience suggests that heparin grafting may play a role in preventing circuit clotting. For this reason, a new cycle of EBP with oXiris filter in CVVHDF mode with RCA is started. The treatments were completed after 72 h without complications. TMP remained in the range with no alarms. After this acute phase, the patient's general condition gradually improves. During the entire ICU stay, this patient underwent EBP with high cutoff membrane (SepteX, *n* = 1), nonselectively adsorbed high-flux hemodiafilter with heparin (oXiris, *n* = 7), and standard high-flux hemodiafilter (AN69ST150, *n* = 14). CRRT treatment was stopped 14 days before discharge from the ICU. Prescriptions are presented in Table 3.

During hospitalization in the ICU, blood, urine, and bronchial aspirate cultures are positive for *Klebsiella oxytoca*, extended spectrum beta-lactamase (ESBL) producing *Escherichia coli*, *Staphylococcus aureus*, *Pseudomonas aeruginosa*, and *Candida albicans*. During the same period, the patient was treated with ceftazidime, piperacillin tazobactam, vancomycin, linezolid, meropenem, and rifaximin. The patient is discharged on spontaneous breathing, without amines and with negative culture and blood tests: white blood cell (WBC) count 7.5 × 10/L, CRP 39.5 mg/L, PCT 0.25 ng/mL.

Written informed consent was obtained from the patient.

## 3. Discussion

In this case report, we focused on the use of sequential EBP in a critically ill COVID-19 patient who worsened rapidly after admission to intensive care. More interesting than the specific case presentation is the self-critical and analytical evaluation of EBP practice at the bedside of a COVID-19 patients with multiple-organ failure treated for more than 6 months in the ICU. This objective assessment was made possible using a web-based registry platform that facilitates data collection and enables continuous monitoring of outcomes and local practice. Critically ill COVID-19 patients are admitted to intensive care for respiratory failure, which may evolve into a multiorgan dysfunction syndrome requiring extracorporeal organ support. A rationale for the use of blood purification strategies to prevent organ damage, protect against further insult, and support organ dysfunction in severe COVID-19 patients characterized by AKI, hyperinflammation, and severe hypercoagulability has been suggested. Dynamic assessment of instantaneous organ function or functional reserve (or adaptability) is used in intensive care to define the patient's disease trajectory and predict the development of complications. These assessments are often challenging to be performed in critically ill patients and speculations about the adaptability of the patient's organs are mostly hypotheses rather than objective features. Therefore, the initiation of EBP is currently based on physicians' perception of its potential usefulness rather than protocols and guidelines.

Given the inflammatory nature of COVID-19, in this case report, we considered biomarkers of systemic inflammation to guide EBP indications, in addition to clinical markers of homeostasis, imbalance, multiorgan dysfunction (e.g., hemodynamic instability and hypoxia), and organs adaptability (e.g., furosemide stress test). In the patient presented here, we used CRP, PCT, and ferritin as markers of systemic inflammation that may have contributed to and sustained kidney injury and lung failure through kidney–lung crosstalk. With this rationale, we excluded the use of cartridges that could only target the inflammatory markers and we decided to prescribe membranes for EBP to support organ function (e.g., standard high-flux acrylonitrile membranes) and/or restore immune homeostasis (e.g., high cutoff or oXiris membranes). However, difficulties have been reported in evaluating the efficacy of treatments for which immunomodulation is the main indication. There is currently a lack of agreement on which biomarkers should be used to guide these treatments' initiation and dynamic readjustment.

There is no evidence of the parameters that should be adopted to monitor the efficacy of EBP. No biological markers (neither inflammatory mediators nor specific plasmatic solutes that quantify organ dysfunction) are recognized today as valuable tools for quantifying treatment efficacy and predicting patient response and outcomes. Most clinicians identify rapid recovery from hemodynamic instability and improvements in oxygenation index as markers to recognize patients who appear to benefit from EBP. Overtime variation in the score exploring multiorgan dysfunction (e.g., SOFA or SAPS II) was particularly useful in this patient to monitor his clinical condition and modify EBP therapy prescriptions, not only in terms of “membrane choice” but also in terms of “dose” of extracorporeal treatments.

The dose of EBP treatment must be constantly monitored to ensure appropriate clearance of target molecules. Undertreatment of EBP at the patient's bedside is often assumed.

Unquantifiable membrane fouling, unexpected downtimes, or blood recirculation can reduce the efficiency of a treatment that is only apparently performed safely and effectively in the ICU. In this case report, the unjustified increase in serum creatinine detected by the website registry during EBP treatment is undoubtedly an expression of a mismatch between the rate of solute generation and its extracorporeal clearance, suggesting the occurrence of membrane fouling that may have reduced transmembrane creatinine removal. This phenomenon is unrecognized in most ICU treatments and can lead to a detrimental disconnect between “the treatment actually applied” and “the outcomes observed by the patient.” It is impossible to draw results or conclusions from clinical associations between treatment and outcomes in these terms, since no specific data are collected on the current effective dose delivered, either in bedside practice or in this case report. The lack of information on membrane fouling is of particular interest considering its high frequency and clinical relevance in COVID-19 patients, for whom EBP with RCA is usually performed and in whom the adoption of a membrane with a heparin graft represents an exciting attempt to reduce local clotting activation further and preserve membrane clotting. oXiris was preferred in this patient over standard high-flux membranes, even without immunomodulatory indications, to take advantage of its internal surface with a heparin graft, potentially preventing filter clotting. Circuit clotting in this paper was defined by the increase in TMP identified by the CRRT machine during treatment. It occurred only in two episodes during the entire EBP treatment period of 28 prescriptions with a clotting rate of 7%, which is below the literature average. Interestingly, the data recorded in the website registry allow us to speculate on the efficacy of EBP in terms of membrane choice, dose, and transmembrane clearance, on the efficacy of anticoagulation strategies adopted, and finally on the occurrence of membrane fouling. All these variables, and especially their interaction in defining the overall strategy adopted for EBP, are usually complex to manage at the patient's bedside. Using a systematic tool to objectively record all these variables could represent a significant improvement for continuous self-assessment and readjustment of local protocols.

Finally, the case presented in this article describes a complex COVID-19 patient with bacterial superinfection. In the case of COVID-19 with suspected or confirmed superimposed Gram-negative bacterial infections, using oXiris could be helpful in early stages to ensure the adsorption of endotoxins [6]. However, source control and antibiotic treatment must be recognized as the only effective etiological therapy that should always be provided to septic patients without delay. In septic patients receiving CRRT, antibiotics should be dosed at therapeutic levels, although many barriers prevent this. Consequently, antibiotics are often underdosed [7]. Antibiotic dosing needs to be readjusted to achieve and maintain adequate plasma concentrations of antimicrobials [8], considering that their pharmacokinetics (PK) changes during sepsis and EBP in critically ill patients [9]. The current approach followed in our hospital is to refer to the Sanford Guide to optimize antimicrobial prescribing during CRRT and EBP [10]. Furthermore, automatic calculation of extracorporeal antibiotic clearance from the website registry (considering the specific hemofilter used and treatment flows prescribed) can further facilitate achieving PK/PD goals by increasing drug dosage during EBP.

## 4. Conclusion

EBP is often adopted in the ICU. Integrated and user-friendly technology has helped to spread these treatments worldwide, even during COVID-19 pandemic.

Improvements in technology and product availability can enable the ICU physician to prescribe the most appropriate therapy at the right time for the specific patient.

However, uncertainties remain regarding the adoption and practical use of these treatments. At present, it is impossible to recognize any practical suggestions for simplifying the clinical use of EBPs at the patient's bedside. The same applies to clinical patient monitoring and extracorporeal treatment. No specific markers have been identified to prognosticate the patient's responsiveness and outcome. Finally, several uncertainties still exist on the approach to be used to ensure PK/PD antimicrobial targets in septic patients undergoing EBP. New technologies based on computer-based communication can help clinicians maintain control over technical and clinical variables that critically influence patient outcomes and periodically reassess local strategies and practices for EBP.

## Figures and Tables

**Table 1:** Patient parameters describing organ function in the first 13 days after ICU admission.

**Day**	**1**	**3**	**4**	**5**	**6**	**7**	**8**	**9**	**10**	**11**	**12**	**13**
MAP (mmHg)	70	70	86.6	86.5	89.9	93.2	89.9	89.9	89.9	93.2	89.9	89.9
NE (mcg/kg/min)	No	0.15	0.15	No	No	No	No	No	No	No	No	No
Pinsp/PEEP (cm/H_2_O)	26/15	18/15	22/15	22/15	22/15	22/15	22/15	22/15	22/15	22/15	22/15	22/15
PaO_2_/FiO_2_	189	210	217.8	238.4	240	220	240	197.7	202.9	204.6	210.6	198.4
Lac (mmol/L)	1.77	1.77	1.67	1.3	1.54	1.43	1.49	1.35	1.41	1.05	1.14	1.42
Hematocrit %	35.9	31	31.5	29.3	29.2	27	22.1	27.4	25.9	27.8	25.9	26.5
Creatinine (mg/dL)	0.82	2.56	4.95	3.31	2.76	2.22	2.4	2.85	4.12	2.45	2.05	2.05
Urea (mg/dL)	36	25	36	40	30	40	60	40	104	40	104	100
Total bilirubin (mg/dL)	1.19	2.5	4.29	5.94	10.86	10.86	13.2	14.86	17	23.96	29.6	29.7
CRP (mg/L)	94.7	304	335	254	254	332.2	335.5	295	290.2	266	222.3	182
PCT (ng/mL)	3.05	3.05	3.89	2.46	2.04	2.52	3.52	3.54	7.45	5.37	5.29	5.55
SOFA score	12	12	12	12	13	13	14	14	14	13	13	14

Abbreviations: CRP, C-reactive protein; FiO_2_, fraction of inspired O_2_; Lac, concentration of blood lactate; MAP, mean arterial pressure; NE, norepinephrine; PaO_2_, partial pressure of oxygen in the arterial blood; Pinsp, inspiratory pressure (cm/H_2_O); PEEP, positive-end expiratory pressure (cm/H_2_O); PCT, procalcitonin; SOFA, sequential organ failure assessment.

**Table 2:** CRRT prescriptions and complications in the first 13 days after ICU admission.

**ICU Day**	**1–3**	**4**	**5**	**6**	**7**	**8**	**9**	**10**	**11**	**12**	**13**
CRRT	No	CVVHD	CVVHDF	CVVHDF	CVVHDF	CVVHDF	CVVHDF	CVVHDF	CVVHDF	CVVHDF	CVVHDF
Filter		SepteX	oXiris	oXiris	oXiris	AN69ST	AN69ST	oXiris	oXiris	oXiris	oXiris
Qb (mL/min)		120	150	150	120	110	110	120	130	130	130
Qd (mL/h)		2500	1200	1200	1200	1300	1300	1500	1200	1200	1200
Qr pre (mL/h)		No	0	0	0	0	0	0	0	0	0
Qr post (mL/h)		No	300	300	300	500	500	500	300	300	300
Net-UF (mL/h)		120	180	180	180	150	100	180	180	150	150
Current dose (mL/kg/h)		28.54	32.07	32.07	29.04	30.76	30.25	34.08	30.05	29.75	29.75
Anticoagulation		UFH	Citrate	Citrate	Citrate	Citrate	Citrate	Citrate	Citrate	Citrate	Citrate
Total/ion calcium			2.146	1.772	1.983	2.034	2.203	2.079	2.149	2.022	2.392
Ca compensation			CaCl_2_	CaCl_2_	CaCl_2_	CaCl_2_	CaCl_2_	CaCl_2_	CaCl_2_	CaCl_2_	CaCl_2_
Ca compensation (%)			100	100	100	100	100	100	90	100	100
Complication		Clotting	No	No	No	No	Clotting	No	No	No	No

*Note:* Data from the ARRT registry (http://www.arrt.eu).

Abbreviations: Net-UF, net ultrafiltration; Qb, blood flow; Qd, dialysate flow; Qr, prereplacement flow in predilution; Qr, postreplacement flow in postdilution; UFH, unfractioned heparin.

**Table 3:** CRRT filters and anticoagulation strategies during the entire ICU length of stay.

**Filter**	**SepteX**	**oXiris**	**AN69ST150**
Cycles (*n*)	1	7	14
Anticoagulation	UFH	RCA	RCA

Abbreviations: RCA, regional citrate anticoagulation; UFH, unfractioned heparin.

## Data Availability

Data used for this study are available on the ARRT Registry (http://www.arrt.eu/).
